# Brief report – Telomere length is a poor biomarker to predict 1-year mortality or cardiovascular comorbidity in patients with transcatheter aortic valve replacement

**DOI:** 10.1371/journal.pone.0213250

**Published:** 2019-03-12

**Authors:** Martin Steinmetz, Charlotte Schmitter, Tobias Radecke, Anja Stundl, Georg Nickenig, Christian Schaefer, Nadjib Schahab, Mariuca Vasa-Nicotera, Jan-Malte Sinning

**Affiliations:** 1 Universitätsklinikum Essen, Klinik für Kardiologie und Angiologie, Essen, Germany; 2 Universitätsklinkum Bonn, Medizinische Klinik und Poliklinik II, Bonn, Germany; 3 Universitätsklinikum Frankfurt, Klinik für Kardiologie, Frankfurt, Germany; Klinikum Region Hannover GmbH, GERMANY

## Abstract

**Background:**

Transcatheter aortic valve replacement (TAVR) is a therapeutic option for patients with aortic valve stenosis at increased surgical risk. Telomeres are an established marker for cellular senescence and have served to evaluate cardiovascular diseases including severe aortic valve stenosis. In our study, we hypothesized that telomere length may be a predictor for outcome and associated with comorbidities in patients with TAVR.

**Methods and results:**

We analyzed leucocyte telomere length from 155 patients who underwent TAVR and correlated the results with 1-year mortality and severe comorbidities. The cohort was subdivided into 3 groups according to telomere length. Although a trend for a positive correlation of telomere length with a lower EuroSCORE could be found, telomere length was not associated with survival, aortic valve opening area or cardiovascular comorbidities (peripheral, coronary or cerebrovascular disease). Interestingly, long telomeres were significantly correlated to a reduced left ventricular ejection fraction (LVEF).

**Conclusion:**

In elderly patients with severe aortic valve stenosis, leucocyte telomere length did not predict post-procedural survival. The correlation between long telomere length and reduced LVEF in these patients deserves further attention.

## Introduction

Calcific aortic valve disease (CAVD) has a high prevalence of about 2% among the elderly ≥ 65 years, and is the most common heart valve pathology[1][2]. Chronic inflammatory processes rather than passive degeneration are essential for the morphological changes in the aortic cusps leading to calcification [3][4]. To date, the only therapeutic option is aortic valve replacement either through cardiac surgery or transcatheter aortic valve replacement (TAVR)[1]. Whether biomarkers help to predict the outcomes of patients undergoing TAVR is an ongoing debate.

Telomeres form the caps of chromosomes and shorten with ongoing cellular division and senescence. Hence, telomere attrition is inevitable. Short telomeres correlate with age or increased cell turnover due to inflammation. Telomeres have been analyzed in a variety of conditions and diseases, such as bone marrow transplantation, genetic mutations of the DKC1 gene, inflammatory bowel disease or leukemia[5]. Patients with severe calcific aortic valve disease (CAVD) show a significantly higher cellular senescence and shorter telomere length when compared with control patients without CAVD[6].

In our study, we investigated whether telomere length may correlate with 1-year mortality or cardiovascular comorbidities in patients undergoing TAVR.

## Methods

Samples of 155 patients undergoing TAVR were enrolled after written informed consent in the study that was approved by the local medical ethics committee (Ethikkommission an der Medizinischen Fakultät der Rheinischen Friedrich-Wilhelms-Universität Bonn). DNA samples were obtained from whole blood samples. DNA was extracted with the QIAamp DNA Blood Mini Kit (Qiagen). The isolated DNA was measured with a PEQLAB Nano Drop 2000c spectrophotometer. Telomere length was analyzed with a PCR-based technique that compares telomere repeat sequence copy number to single-copy gene (36B4) copy number in a given sample as published elsewhere[7]. Duplicate DNA samples were amplified in parallel PCR reactions of 0,0768 ng genomic DNA, Takyon Rox SYBR Mastermix blue dTTP and either telomere primers (forward: 5’-GGGTTTGTTTGGGTTTGGGTTTGGGTTTGGGTTTGGGTT-3’; reverse: 5’- GGCTTGCCTTACCCTTACCCTTACCCTTACCCTTACCCT-3’); or 36B4 primers (forward: 5’-CAGCAAGTGGGAAGGTGTAATCC-3’; reverse: 5’-CCCATTCTATCATCAACGGGTACAA-3’). All PCRs were run on a ThermoFisher 7500 Fast Real-Time PCR System (initially 50° C for 2 min and 95°C for 10 min, then 15 s at 95°C, 30 s at 58°C and 30 s at 72° C for 40 cycles). The linear range of the assay was determined by using serially diluted DNA (1,92 ng, 0,384 ng, 0,0768 ng, 0,01536 ng). Both PCR reactions exhibited good linearity across this input range (r^2^ > 0.95). Test samples were checked to be within this range, outliers were diluted as necessary and re-run. 6 study samples (diluted four times), a calibrator sample, and one no-template control sample (all in triplets) were processed per run. The calibrator sample was used to enhance inter-assay comparability.

Continuous variables are expressed as the mean ± standard error mean and analyzed by a two-sided unpaired Student’s t-test. Categorical variables were compared by chi-square test. Survival rates were compared with the log-rank and Breslow test. Statistical analyses were performed with Prism 6.0 (Graphpad Software). A p-value < 0.05 was considered statistically significant.

## Results

**Fig 1:**
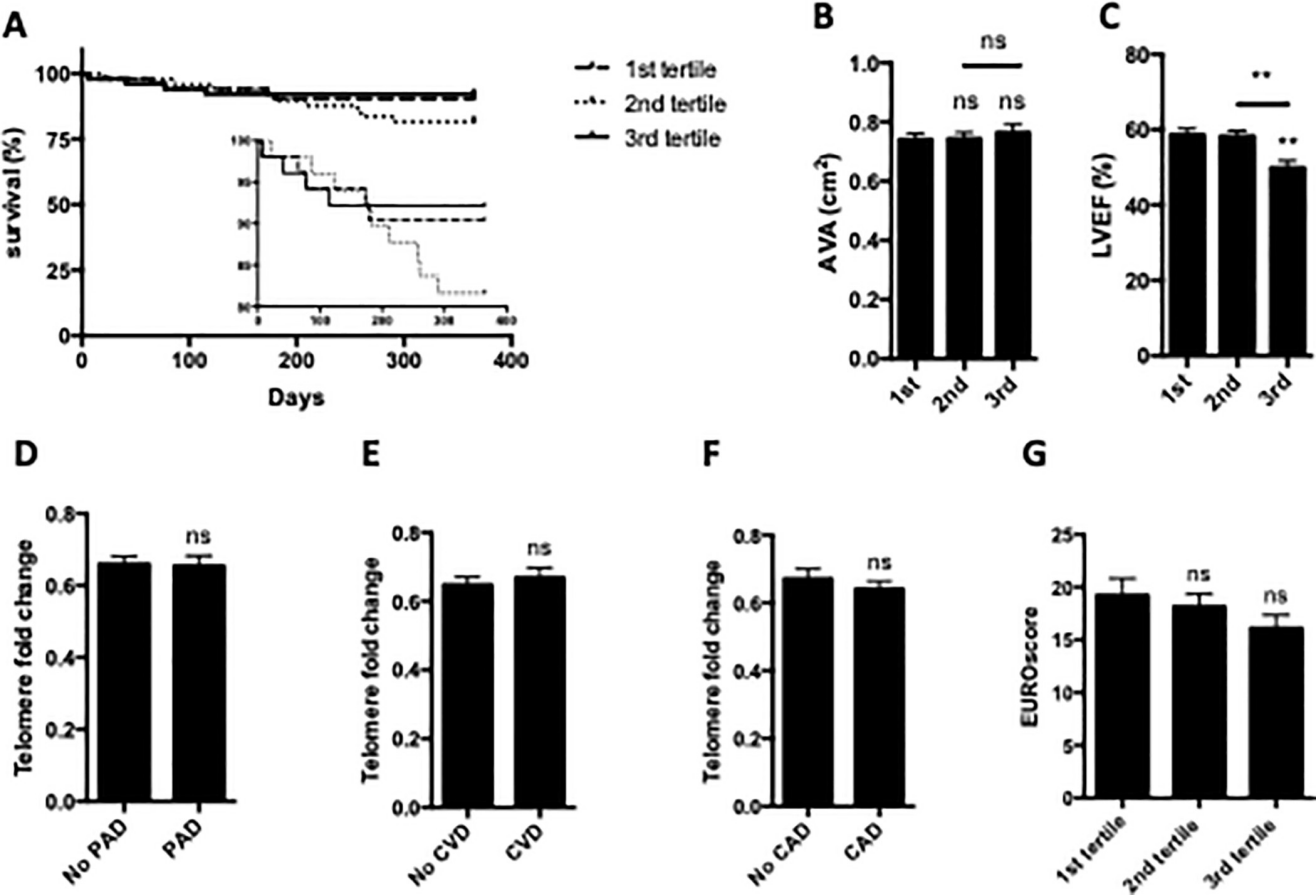
**A)** Kaplan-Meyer survival curves of TAVR patients did not show a significant correlation between relative telomere length and mortality (1^st^ tertile: shortest telomeres, 2^nd^ tertile: median telomeres, 3^rd^ tertile: longest telomeres). **B)** The aortic valve opening area (AVA) was similar in all tertiles. **C)** Left ventricular ejection fraction was significantly reduced in the patients’ tertile with longest telomeres. **D)** Telomere length was not significantly different between TAVR patients without or with peripheral artery disease (PAD), **E)** patients without or with cerebrovascular disease (CVD), or **F)** without or with coronary artery disease (CAD). **G)** Telomere length did not significantly correlate with the EUROscore; ns = not significant, ** p≤0.01, n = 155.

Baseline characteristics of patients before TAVR that were grouped according to their telomere length, i.e. from shortest (1^st^ tertile) to longest telomere length (3^rd^ tertile). Baseline characteristics are listed in **Table 1**. 30-day and 1-year survival were not significantly different between the 3 groups with short, intermediate and long telomere length (30-days: 1^st^ tertile 98.1%, 2^nd^ tertile 98.1%, 3^rd^ tertile 98.0%; 1-year: 1^st^ tertile 90.4%, 2^nd^ tertile 76.9%, and 3^rd^ tertile 92.2%; all p>0.05) (**Fig 1A**).

**Table 1:** Baseline characteristics. Cardiovascular disease, risk profile and echocardiographic parameters are displayed for all patients (total) and split in tertiles referring to telomere length.

	Total	1^st^ tertile (<0,5202)	2^nd^ tertile (0,5202 -0,7232)	3^rd^ tertile (>0,7232)	p
	N = 155	N = 52	N = 52	N = 51	
**Age (years)**	80.53±5.87	80.87±5.92	80.65±5.88	80.06±5.77	0.52
**Sex**					0.65
Male	76 (49.0%)	22 (42.3%)	31 (59.6%)	23 (45.1%)	
Female	79 (51. 0%)	30 (57.7%)	21 (40.4%)	28 (54.9%)	
**Coronary artery disease**	86 (55.5%)	29 (55.8%)	33 (63.5%)	24 (47.1%)	0.49
1-vessel disease	33 (38.4%)	10 (34.5%)	12 (36.4%)	11 (45.8%)	
2-vessel disease	16 (18.6%)	3 (10.3%)	9 (27.3%)	4 (16.7%)	
3- vessel disease	37 (43.0%)	16 (55.2%)	12 (36.4%)	9 (37.5%)	
**Peripheral artery disease**	47 (30.3%)	14 (27.5%)	19 (36.5%)	14 (27.5%)	0.94
**Cerebrovascular disease**	51 (32.9%)	14 (26.9%)	18 (34.6%)	19 (37.3%)	0.50
***EuroSCORE***					0.11
<20	100 (64.5%)	32 (61.5%)	33 (63.5%)	35 (68.6%)	
≥20	55 (35.5%)	20 (38.5%)	19 (36.5%)	16 (31.4%)	
**Left ventricular ejection fraction (%)**	55.6±12.7	58.6±11.8	58.3±10.7	49.9±13.6*	<0.05
**Aortic valve opening area (cm**^**2**^**)**	0.75±0.01	0.74±0.02	0.74±0.02	0.76±0.03	0.70
**Mean pressure gradient (mmHg)**	45.3±1.3	44.0±2.4	46.0±2.2	42.3±2.1	0.62
**Type 2 Diabetes mellitus**	36 (23.2%)	12 (23.1%)	13 (25.0%)	11 (21.6%)	0.87
**Smoking**	45 (29.0%)	13 25.0%)	14 (26.9%)	18 (35.3%)	0.45
**Hypertension**	129 (83.2%)	42 (80.8%)	46 (88.5%)	41 (80.4%)	0.55
**Family history**	12 (7.7%)	3 (5.8%)	6 (11.6%)	3 (3.9%)	0.48
**Total cholesterol (mg/dL)**	169.3±4.1	173.9±8.1	159.3±7.0	174.6±6.1	0.19
LDL	102.7±3.3	106.2±6.6	94.86±5.5	107.0±4.9	0.15
HDL	51.72±1.2	53.3±2.4	47.74±2.0	54.06±2.0	0.08

* p≤0.05, n = 155

Telomere length showed no significant correlation with aortic valve opening area (**Fig 1B**: AVA; 1^st^ tertile: 0.78 cm^2^, 2^nd^ tertile: 0.73 cm^2^; 3^rd^ tertile: 0.75 cm^2^). However, long telomere length was significantly associated with a reduced left ventricular ejection fraction at baseline (**Fig 1C**: LVEF; 1^st^ tertile: 58.6%; 2^nd^ tertile: 58.3%; 3^rd^ tertile: 49.9%; p ≤ 0.05 vs. 1^st^ and 2^nd^ tertile).

Further, we found no overall difference in patients with or without peripheral artery disease, coronary artery disease or carotid artery stenosis when looking at overall telomere length or comparing subgroups in terms of telomere length (**Fig 1D–1F**). Lastly, we compared the EuroSCORE and found no significant difference between short, intermediate and long telomere length (**Fig 1G**: 1^st^ tertile: EuroSCORE 19.2 ± 1.6; 2^nd^ tertile: 18.4 ± 1.3; 3^rd^ tertile: 15.8 ± 1.5; p>0.05).

## Discussion

The results of our retrospective analysis suggest, that telomere length in peripheral blood is not useful as a biomarker to predict outcomes in elderly patients with various co-morbidities undergoing TAVR. We have used an experimental setup using quantitative PCR with a reference gene[7], and creating a very good inter-assay comparability by adding a calibrator sample. The advantage of this approach is the very easy and rapid analysis with standard quantitative real time PCR. Peripheral, carotid or coronary artery disease in combination with severe aortic valve disease was not associated with further telomere shortening. A single cardiovascular co-morbidity obviously does not significantly promote or inhibit telomere shortening in our cohort. Nevertheless, telomere length might reflect global disease burden. The logistic EuroSCORE comprises variables for organ (dys-)function, active inflammation, or chronic diseases and hence reflects overall disease burden with a cardiovascular pronunciation. We found a non-significant tendency that it was negatively correlated to telomere length, suggesting that a higher degree of frailty may be linked to shorter telomeres in an adequately powered study. The contribution of a single risk factor or disease probably cannot be extrapolated in patients with complex co-morbidities, because—depending on the specific disease—cellular activation and turnover may have even opposite effects on telomere length and senescence. In a recent study by Kurz and co-workers, calcific aortic valve disease but not coronary artery disease was linked to shorter telomeres[6]. On the contrary, in patients with premature myocardial infarction, telomere length was inversely correlated with disease burden[8][9]. In ApoE/TERC-deficient mice with significantly disturbed telomerase activity and shorter telomere, atherogenesis progress more slowly when compared to ApoE-deficient controls[10][11], probably a consequence of immuno-incompetence. Thus, it is finally not clear whether short telomeres lead to or result from pronounced atherogenesis.

Although no data on valvular cardiomyopathy and telomere attrition exist, studies of patients with congestive heart failure[11] or ischemic heart failure such as CORONA[12] have conclusively shown that congestion and mortality are inversely correlated with leucocyte telomere length. Surprisingly, left ventricular ejection fraction was significantly diminished in the patients with the longest telomere length in our study. This is a puzzling finding that has not been described in earlier studies in mice or men. In mouse models of myocardial infarction and remodeling, leucocyte mobilization and prolonged inflammation in the myocardium is associated with decreased ventricular function[13]. Because telomere attrition mirrors cell turnover and replication, one may speculate that our patients with longer telomeres might be more immuno-competent, which might negatively influence ventricular remodeling upon severe aortic valve stenosis. Importantly, we investigated circulating leucocyte telomere length and do not have information on cardiomyocyte telomere length, which might have added necessary information to understand the unpredicted lower left ventricular function. It may well be possible that a significant telomere shortening is found in other cell types and compartments, e.g. valvular endothelial or interstitial cells.

Of note, the main reasons for death following TAVR are non-cardiac origin such as sepsis/infection, stroke or cancer. Cardiac reasons are heart failure and arrhythmia[14]. Mortality was very low in the 30-days and 1-year follow up and equally distributed between the 3 groups. Leucocyte telomere length could not predict survival.

Taken together, telomere length as a marker for senescence is not significantly correlated to survival or single comorbidities in patients with severe aortic valve stenosis who underwent TAVR.

## Supporting information

S1 Data Raw data can be found in “raw data PONE”. (XLSX)
